# Endoplasmic reticulum stress and organelle crosstalk: Core mechanisms and therapeutic targets in diabetic kidney disease

**DOI:** 10.1016/j.metop.2026.100510

**Published:** 2026-10-14

**Authors:** Yanhong Gao, Huijun Wu, Henglan Wu, Yinfeng Guo, Huanqing Gao

**Affiliations:** a Department of Cardiometabolic Medicine, Renmin Hospital, Wuhan University, Wuhan, 430000, China; b Department of Nephrology, Affiliated Hospital of Jiaxing University (The First Hospital of Jiaxing), Jiaxing, Zhejiang, 314000, China

**Keywords:** Diabetic kidney disease, Endoplasmic reticulum stress, Unfolded protein response, Lipotoxicity, Calcium signaling, Mitochondria-associated membranes

## Abstract

Diabetic kidney disease (DKD) remains the leading global cause of end-stage renal disease, with persistent residual renal damage even after standard clinical interventions including SGLT2 inhibitors and GLP-1 receptor agonists. Accumulating preclinical evidence indicates that sustained endoplasmic reticulum (ER) dysfunction may act as an important integrating mechanism contributing to renal cellular injury in DKD, beyond its canonical function in protein folding, although direct clinical evidence in human DKD remains limited. This review examines how interacting diabetic metabolic and inflammatory stressors, including glucotoxicity, lipotoxicity, advanced glycation end products, and chronic inflammation, disrupt ER homeostasis and promote persistent ER stress. Long-term ER perturbation induces a pathogenic shift of the unfolded protein response (UPR) from adaptive homeostasis maintenance toward pro-apoptotic and pro-inflammatory signaling, alongside disordered lipid biosynthesis and Ca^2+^ dyshomeostasis. These three core pathological alterations jointly facilitate podocyte and tubular epithelial cell apoptosis, sterile renal inflammation and progressive interstitial fibrosis, while altered mitochondria-associated ER membrane (MAM) dynamics may amplify organelle injury and exacerbate disease progression. We further discuss multiple therapeutic strategies targeting ER homeostasis restoration, such as chemical molecular chaperones, selective UPR pathway modulators, SREBP lipogenic inhibitors, SERCA calcium pump activators and MAM stabilizers. We also discuss the potential contribution of ER homeostasis to the renoprotective effects of SGLT2 inhibitors and GLP-1RAs. Overall, ER dysfunction may contribute to DKD pathogenesis and represents a potential therapeutic target, although its mechanistic significance in human DKD requires further investigation.

## 1 Introduction

Diabetic kidney disease (DKD) is the leading cause of end-stage renal disease (ESRD) globally, presenting a significant clinical and economic burden. The introduction of sodium-glucose cotransporter-2 (SGLT2) inhibitors and glucagon-like peptide-1 receptor agonists (GLP-1RAs) has transformed care, providing substantial cardiorenal protection [1], [2], [3]. Nonetheless, a substantial residual risk remains, as numerous patients continue to experience a gradual decrease in glomerular filtration rate (GFR) and advancing albuminuria despite optimal treatment. This underscores the need to better understand the cellular and molecular mechanisms underlying renal injury and to identify therapeutic targets that may provide additional protection.

The endoplasmic reticulum (ER), a dynamic organelle crucial for protein homeostasis, lipid biosynthesis, and calcium storage, is central to maintaining cellular homeostasis [16], [17], [18]. In renal cells with high synthetic and secretory demands, including podocytes and proximal tubular epithelial cells, maintaining ER homeostasis is essential for cellular integrity [19], [20]. The unfolded protein response (UPR) is a key adaptive mechanism that preserves ER proteostasis in response to cellular stress. In diabetes, persistent metabolic and inflammatory stress can disrupt ER homeostasis and impair its capacity to maintain protein, lipid and Ca^2+^ balance [20], [21], [22], [23], [24].

Consequently, the objective of this review is to critically examine the role of ER dysfunction as an integrative component in the pathogenesis of DKD. We first introduce the physiological and adaptive functions of the UPR. We then discuss the major diabetic stressors that promote persistent ER stress, including glucotoxicity, lipotoxicity, AGE–RAGE signaling, oxidative stress, inflammation, and metabolic disturbances associated with hyperinsulinemia and insulin resistance. Next, we examine the major manifestations and consequences of ER dysfunction, including maladaptive UPR signaling, altered ER lipid metabolism, Ca^2+^ dyshomeostasis, and ER–mitochondrial crosstalk, with particular emphasis on their contribution to renal cellular injury and fibrosis. Finally, we review emerging ER-targeted therapeutic strategies and discuss the potential relevance of current renoprotective therapies to ER and organelle homeostasis. By delineating the link between diabetic metabolic stressors, ER dysfunction, and progressive renal injury, this review highlights the potential therapeutic value of targeting ER dysfunction in DKD.

## 2 Literature search and study selection

This narrative review was informed by a structured literature search of PubMed/MEDLINE, Web of Science, and Scopus for English-language articles published between January 2000 and May 2025. The database search was conducted on 24 May 2025. The literature search was subsequently updated using the same search terms and inclusion criteria, covering the period from May 2025 to October 2026. The final updated search was conducted on 9 October 2026. Search terms combined DKD-related keywords (“diabetic kidney disease,” “diabetic nephropathy,” and “DKD”) with terms related to ER stress and its major mechanisms, including “endoplasmic reticulum stress,” “unfolded protein response,” “UPR,” “IRE1,” “PERK,” “ATF6,” “lipotoxicity,” “glucotoxicity,” “calcium homeostasis,” “SERCA,” and “mitochondria-associated membranes.” Relevant therapeutic terms, including “chemical chaperones,” “UPR modulators,” “SGLT2 inhibitors,” and “GLP-1RAs,” were also considered. Search results were reviewed for relevance, and potentially relevant full-text articles were evaluated. Original research, preclinical and clinical studies, and relevant reviews were included, whereas conference abstracts, case reports, and studies focused exclusively on non-renal diabetic complications were excluded. Priority was given to original studies, DKD-specific models, human or clinical evidence where available, and mechanistically informative reports.

## 3 The unfolded protein response: Adaptive ER homeostasis

The UPR is an adaptive signaling network that maintains ER proteostasis in response to disturbances in protein folding and ER homeostasis. Its three main branches—inositol-requiring enzyme 1α (IRE1α)-X-box binding protein 1 (XBP1), protein kinase R-like ER kinase (PERK)-eIF2α-activating transcription factor 4 (ATF4), and activating transcription factor 6 (ATF6)—act coordinately to restore ER proteostasis and promote cell survival [25].

The endoribonuclease activity of IRE1α promotes the unconventional splicing of XBP1 mRNA to generate the active transcription factor XBP1s [26]. XBP1s increases the expression of genes encoding ER chaperones, including glucose-regulated protein 78 (GRP78)/binding immunoglobulin protein (BiP), as well as foldases and components of ER-associated degradation (ERAD). At the same time, PERK phosphorylates eukaryotic initiation factor 2α (eIF2α), thereby transiently reducing global protein translation and alleviating the ER protein-folding burden [27]. Although this attenuates general protein synthesis, it selectively promotes the translation of specific transcripts, including ATF4, which regulates amino acid metabolism and antioxidant responses. The full-length ATF6 is transported to the Golgi, where it undergoes proteolytic activation, leading to increased expression of chaperones and ERAD-related genes [28].

Together, these three UPR branches coordinate protein synthesis, folding, degradation, and cellular adaptation to maintain ER homeostasis. Under transient or mild ER stress, this adaptive response can restore ER function and support cell survival.

## 4 The diabetic milieu: Metabolic triggers of ER stress

The diabetic kidney is exposed to multiple, interacting metabolic and inflammatory stressors, including chronic hyperglycemia, excess free fatty acids, advanced glycation end products (AGEs), oxidative stress, and pro-inflammatory cytokines. These insults perturb ER homeostasis through complementary mechanisms and collectively promote ER stress in renal cells. The resulting UPR is initially adaptive; however, when metabolic stress persists, the balance may shift toward maladaptive IRE1α-, PERK-, and ATF6-associated signaling in a cell- and context-dependent manner, thereby coupling ER dysfunction to inflammation, apoptosis, and fibrogenic responses in DKD (Fig. 1).

**Fig. 1:**
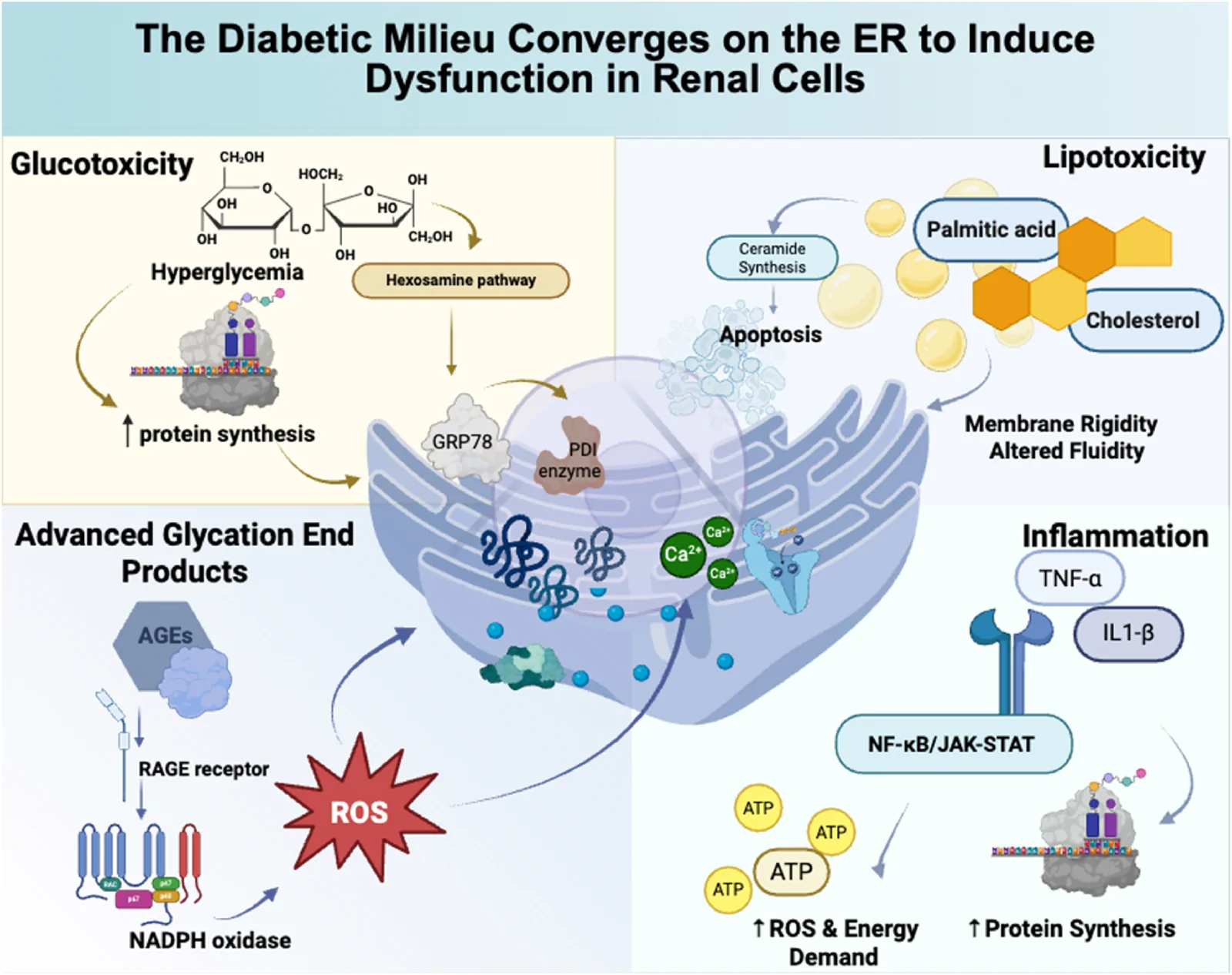
Multiple pathways of the diabetic milieu converge to induce ER dysfunction in renal cells. Four major diabetic insults — glucotoxicity (orange), lipotoxicity (red), AGEs (brown), and inflammation (purple) — target distinct aspects of ER biology in renal cells. Their combined action leads to the three hallmarks of ER dysfunction: proteostasis collapse, lipid bilayer stress, and Ca^2+^ dyshomeostasis.

### 4.1 Glucotoxicity: increased proteostatic demand and maladaptive UPR signaling

Chronic hyperglycemia perturbs ER proteostasis through several convergent mechanisms. First, increased glucose availability enhances extracellular matrix synthesis, secretory, and membrane proteins, thereby increasing the protein-folding burden on the ER. In mesangial and tubular cells, for example, high glucose promotes the synthesis of type IV collagen and other matrix components [29], [30]. Second, excess glucose flux through the hexosamine biosynthetic pathway increases UDP-N-acetylglucosamine availability and alters protein O-GlcNAcylation. Aberrant O-GlcNAc modification of ER-resident proteins, including molecular chaperones and protein disulfide isomerases, may impair folding capacity and promote the accumulation of incompletely folded proteins [31], [32].

Accumulation of misfolded proteins activates the UPR as an initial adaptive response to restore ER proteostasis. However, when hyperglycemic stress persists, sustained UPR activation may become maladaptive. In experimental diabetic nephropathy, impaired podocyte XBP1 signaling promotes an ATF6-dependent maladaptive ER-stress response and contributes to podocyte dysfunction [33]. Recent experimental studies further show that hyperglycemic renal stress is associated with activation of PERK–eIF2α–ATF4–CHOP signaling and that excessive UPR activation can induce renal cell apoptosis and inflammatory injury [32], [34]. Thus, chronic hyperglycemia may shift the UPR from an initially protective response toward cell-injurious signaling when ER stress remains unresolved.

### 4.2 Lipotoxicity: ER membrane stress and UPR activation

Lipotoxicity can directly perturb ER membrane structure and function. In insulin-resistant states, increased circulating saturated free fatty acids (SFAs, such as palmitate), enhanced renal lipid uptake, and dysregulated de novo lipogenesis can alter cellular lipid composition [35]. Excess SFAs and cholesterol increase ER membrane saturation and rigidity, thereby disrupting membrane-associated proteins involved in Ca^2+^ handling, protein translocation, and lipid metabolism [36]. This membrane disturbance, termed lipid bilayer stress, constitutes a distinct source of ER stress.

Importantly, lipid-induced UPR activation does not necessarily require accumulation of misfolded proteins. Increased membrane lipid saturation can activate IRE1α and PERK through their transmembrane domains, providing a protein-misfolding-independent mechanism of UPR activation [37]. In parallel, SFAs such as palmitate promote ceramide accumulation and other lipotoxic responses [38]. In podocytes, palmitate exposure induces ER stress markers, including GRP78 and CHOP, and promotes apoptosis. Thus, persistent lipotoxic stress can couple ER membrane dysfunction to maladaptive UPR signaling, Ca^2+^ dysregulation, and mitochondrial oxidative stress, contributing to podocyte and tubular injury in DKD [39].

### 4.3 AGE–RAGE signaling, oxidative stress, and inflammation

AGEs and chronic inflammatory signaling further amplify ER stress in the diabetic kidney. AGEs are formed through non-enzymatic modification of proteins, lipids, and nucleic acids, and their binding to RAGE activates NADPH oxidases and increases ROS production [40], [41], [42], [43]. Excessive ROS disrupts ER redox homeostasis, impairs protein folding, and promotes ER Ca^2+^ depletion, thereby increasing ER stress and activating the UPR [44].

Pro-inflammatory cytokines, including IL-6, IL-1β, and tumor necrosis factor-α (TNF-α), further exacerbate ER stress through inflammatory and redox-sensitive pathways. TNF-α can enhance protein synthesis while inhibiting proteasomal degradation, increasing the protein-processing burden on the ER [45], [46]. Thus, AGEs and inflammatory cytokines amplify ER dysfunction through ROS, Ca^2+^ dysregulation, and impaired protein quality control, promoting persistent UPR activation.

### 4.4 Hyperinsulinemia and insulin resistance

Hyperinsulinemia and insulin resistance are important components of the diabetic milieu and may influence ER homeostasis through tissue-specific mechanisms. In pancreatic β-cells, increased insulin demand increases protein-folding requirements and activates the UPR to support β-cell adaptation [47]. In podocytes, insulin signaling also contributes to ER homeostasis through the p85–XBP1 pathway, whereas impaired insulin signaling promotes ATF6-dependent maladaptive ER stress and aggravates diabetic nephropathy [33]. Thus, the mechanisms observed in β-cells should not be directly extrapolated to renal cells.

Sustained hyperinsulinemia can impair renal insulin responsiveness by promoting insulin receptor degradation and podocyte insulin resistance [48]. More recently, hyperinsulinemia was shown to promote podocyte injury and albuminuria through a GSK3β–IRS1 insulin-desensitization pathway in experimental models [49]. Insulin signaling also regulates podocyte bioenergetics and mitochondrial dynamics, with prolonged insulin exposure altering mitochondrial morphology and increasing mitophagy [50]. Hyperinsulinemia and insulin resistance may additionally affect renal hemodynamics and sodium handling [51] and, together with dyslipidemia, oxidative stress, and chronic inflammation, may increase renal susceptibility to organelle dysfunction and progressive injury. However, direct evidence that circulating hyperinsulinemia independently activates the renal UPR remains limited. Hyperinsulinemia and insulin resistance may therefore act as metabolic and signaling modifiers that amplify renal ER dysfunction rather than established primary initiators of renal ER stress.

## 5 Core manifestations of ER dysfunction: from stress to homeostatic collapse

The transition from ER stress to ER dysfunction represents an important shift in the pathogenesis of diabetic kidney disease (DKD). This transition reflects a shift from adaptive ER stress responses toward loss of ER homeostasis and maladaptive signaling that contributes to cellular injury. The major manifestations of this dysfunction can be grouped into three main areas: dysregulation of the unfolded protein response (UPR), disruption of lipid metabolism, and Ca^2+^ dyshomeostasis [52], [53].

### 5.1 UPR dysregulation

In the initial or mild stages of DKD, this adaptive UPR may provide temporary resilience [54]. However, chronic exposure to the diabetic milieu can exceed the adaptive capacity of the UPR, favoring a transition toward maladaptive signaling. This transition is particularly evident in the IRE1α and PERK pathways.

Under prolonged ER stress, IRE1α′s activity changes from adaptive XBP1 splicing to Regulated IRE1α-Dependent Decay (RIDD) [55]. RIDD promotes the degradation of ER-associated mRNAs, including transcripts encoding secreted proteins and components of the ER, thereby compromising cellular function [56]. More importantly, hyperactivated IRE1α oligomers recruit TNF receptor-associated factor 2 (TRAF2), forming a signaling platform that activates apoptosis signal-regulating kinase 1 (ASK1) and its target, c-Jun N-terminal kinase (JNK) [57]. JNK phosphorylates and inactivates anti-apoptotic B-cell lymphoma 2 (Bcl-2) proteins while activating pro-apoptotic Bim and Bcl-2-associated death promoter (BAD), thereby promoting mitochondrial outer membrane permeabilization and apoptosis. At the same time, the chronic PERK-ATF4 axis sustains the expression of the transcription factor C/EBP homologous protein (CHOP) [58]. CHOP is a major mediator of ER stress-induced apoptosis. It suppresses the transcription of the anti-apoptotic protein Bcl-2 and increases the levels of pro-apoptotic BH3-only proteins such as Bcl-2-interacting mediator of cell death (BIM) and p53-upregulated modulator of apoptosis (PUMA) [59], [60]. CHOP can promote oxidative stress by reducing glutathione levels and enhance DR5 signaling [61]. Importantly, CHOP and JNK signaling converge on mitochondria, forming a potent pro-apoptotic synergy.

Maladaptive UPR signaling also promotes inflammation. The IRE1α-TRAF2 complex can activate IκB kinase (IKK), which promotes NF-κB nuclear translocation and induces the expression of cytokines such as IL-6 and TNF-α [62], [63]. The assembly and activation of the NOD-like receptor family pyrin domain containing 3 (NLRP3) inflammasome have been linked to PERK/ATF4 signaling and changes in cytosolic Ca^2+^ levels. This promotes the maturation of IL-1β and IL-18 by caspase-1. Additionally, ER dysfunction hinders the essential clearance processes of ER-associated degradation (ERAD) and ER-phagy (reticulophagy) [64]. Proteins that are unable to be refolded are retrotranslocated to the cytosol for proteasomal degradation through ERAD. In DKD, the proteasome and autophagy-lysosomal systems frequently exhibit dysfunction, resulting in the toxic accumulation of ubiquitinated protein aggregates that intensify ER stress and trigger inflammatory pathways [65] (Fig. 2). Beyond these canonical mechanisms, epigenetic and RNA-level regulation of ER stress is increasingly recognized in DKD. For instance, lncRNA 153 has been reported to alleviate podocyte ER stress by inhibiting PARP1-mediated ATF3 PARylation and reducing ATF3 transcriptional activity [66]. Thus, maladaptive UPR signaling extends beyond defective proteostasis. IRE1α–JNK and PERK–CHOP signaling directly engage mitochondrial apoptotic pathways, while UPR-associated inflammatory and oxidative responses can further perturb ER membrane function and Ca^2+^ handling.

**Fig. 2:**
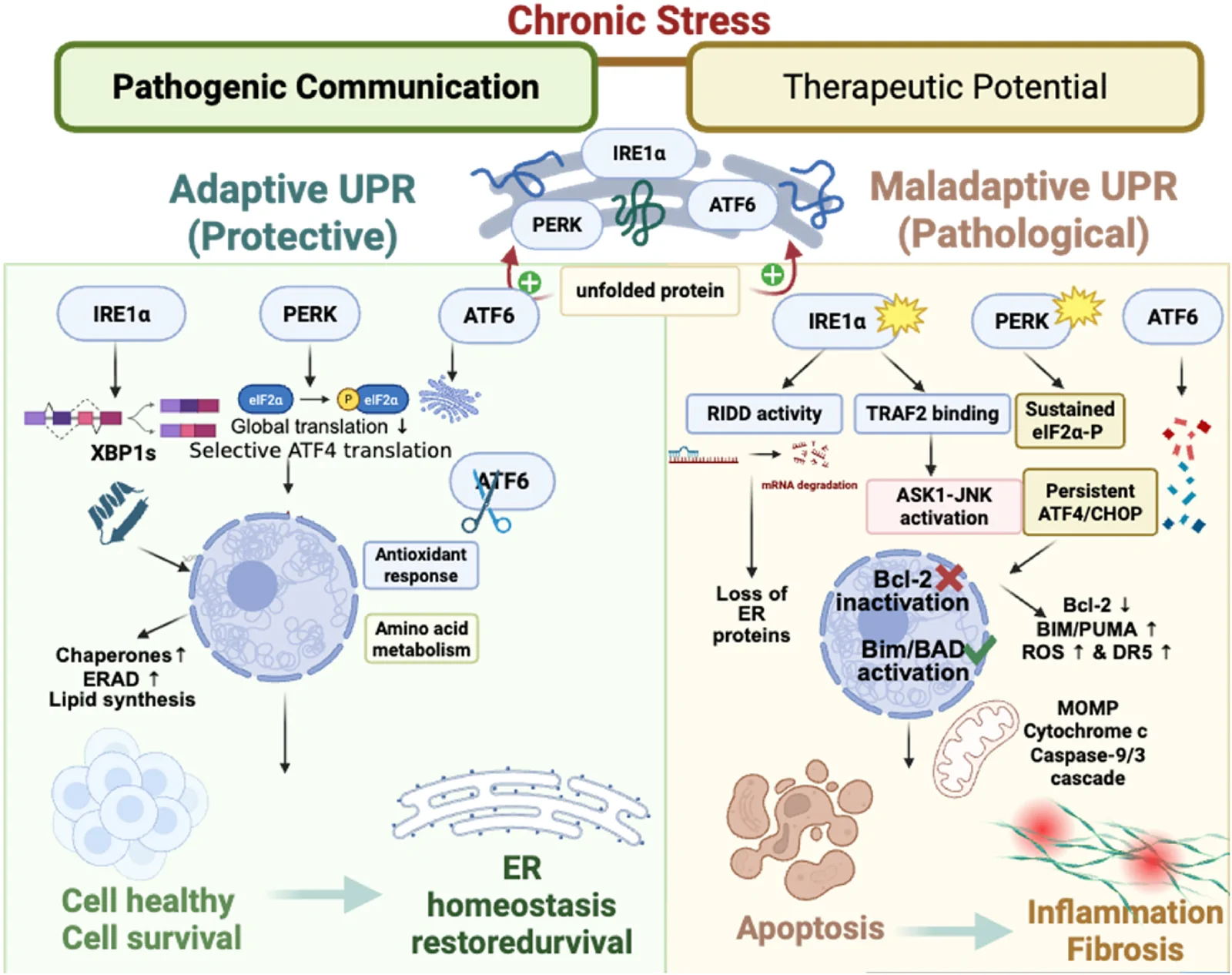
The switch from adaptive to maladaptive UPR signaling in DKD. Under transient stress, the adaptive UPR (left) engages IRE1α–XBP1s, PERK–ATF4, and ATF6 pathways to restore proteostasis and promote cell survival. In the chronic diabetic environment (right), the UPR switches to a maladaptive state characterized by hyperactive IRE1α–RIDD/TRAF2 and sustained PERK–CHOP signaling, which converge to drive apoptosis, inflammation, and fibrosis.

### 5.2 ER lipid metabolism and lipotoxicity

The ER is the principal site of synthesis of phospholipids, cholesterol, and neutral lipids. In DKD, this lipid metabolic function is altered by hyperglycemia, insulin resistance, and ER stress. Hyperactivation of sterol regulatory element-binding proteins (SREBPs), which are master transcription factors for lipogenic genes, is a major contributor to this dysregulation [67]. Insulin resistance, elevated glucose levels, and ER stress can stimulate SREBP-1c and SREBP-2. This promotes transcription of enzymes like acetyl-CoA carboxylase (ACC) and fatty acid synthase (FAS), thereby increasing de novo lipogenesis. Newly synthesized fatty acids are esterified into triglycerides and phospholipids. However, when fatty acid oxidation is impaired (which is common in DKD tubules), this leads to ectopic lipid buildup in kidney cells, thereby contributing to renal lipotoxicity [68], [69]. Podocytes and tubular cells store lipid droplets, and excess lipids are channeled toward lipotoxic pathways, including the generation of diacylglycerol (DAG) and ceramides.

This dysregulation directly changes the lipid makeup of the ER membrane. The membrane becomes stiffer as the saturation index of phospholipids and the cholesterol content increase. This impairs the function of integral membrane proteins. The calcium pump sarco/endoplasmic reticulum Ca^2+^-ATPase (SERCA), which is very sensitive to the lipids around it, and the translocon complex, which is used to bring in proteins, are both examples of this [70]. Altered membrane can also prevent oligomeric protein complexes that help with folding from coming together and working properly. Sphingolipid metabolism is also dysregulated. The buildup of ceramides, especially long-chain ceramides made in the ER, is associated with pro-apoptotic signaling. Ceramides can promote mitochondrial membrane permeabilization, facilitate death receptor signaling complexes, and impair mitochondrial function [71].

### 5.3 Ca^2+^ dyshomeostasis

The ER is the principal intracellular calcium store. It maintains the luminal calcium concentration approximately 10,000 times higher than that in the cytosol. This gradient is important for calcium-dependent chaperones like calnexin and calreticulin to work. It also serves as a place for signal transduction. This tightly regulated system is disrupted in DKD. There are two main ways that enhanced calcium leak happens: through inositol 1,4,5-trisphosphate receptors (IP3Rs) and ryanodine receptors (RyRs) [72]. Oxidative changes caused by diabetic ROS, such as S-nitrosylation and carbonylation, and direct contact with pro-apoptotic factors like Bcl-2-associated death promoter (BAD) make these channels more sensitive [73], [74].

At the same time, the reuptake of Ca^2+^ is impaired. Diabetic stress can impair the sarco/endoplasmic reticulum calcium ATPase 2b (SERCA2b), which mediates Ca^2+^ reuptake into the ER [75]. Nitric oxide (NO)-mediated nitrosylation, direct oxidation, and interaction with misfolded proteins such as GRP78 all impair its activity [76]. Consequently, ER Ca^2+^ stores become depleted, resulting in sustained elevation of cytosolic Ca^2+^.

Cytosolic Ca^2+^ overload provides a direct link between ER dysfunction and mitochondrial injury. Excess cytosolic Ca^2+^ is transferred to mitochondria at mitochondria-associated membranes (MAMs) and taken up through the mitochondrial calcium uniporter (MCU), promoting mitochondrial permeability transition pore opening, loss of membrane potential, impaired ATP production, and cytochrome *c* release [77], [78]. Mitochondrial Ca^2+^ overload also increases ROS generation, which can further oxidize ER proteins and Ca^2+^ -handling machinery, thereby aggravating ER Ca^2+^ leakage and UPR activation [79]. Thus, disruption of ER Ca^2+^ homeostasis can initiate a bidirectional ER–mitochondrial feedback loop that amplifies organelle dysfunction and contributes to apoptosis and inflammatory signaling in DKD (Fig. 3).

**Fig. 3:**
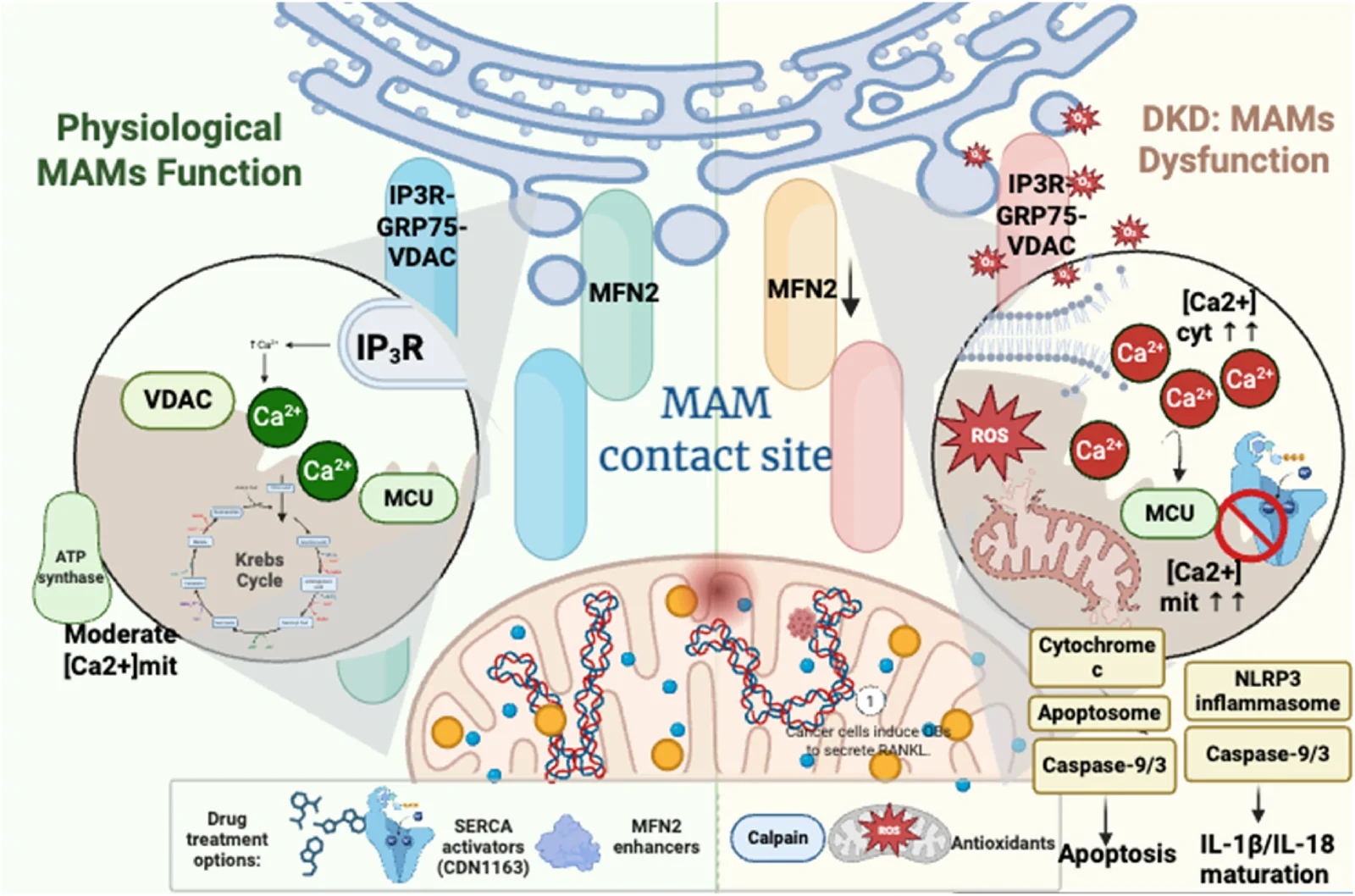
Altered ER–mitochondria contact sites (MAMs) in DKD may amplifie cellular injury. In physiological conditions (left), MAMs facilitate regulated Ca^2+^ transfer, supporting mitochondrial ATP production. In DKD (right), downregulation of tethering proteins (e.g., MFN2), ER Ca^2+^ leak, and SERCA dysfunction cause cytosolic and mitochondrial Ca^2+^ overload. This triggers mitochondrial depolarization, mPTP opening, excessive ROS production, cytochrome *c* release, and activation of apoptotic and inflammatory pathways, establishing a vicious cycle of injury.

### 5.4 Integrated ER–mitochondrial crosstalk in DKD

Collectively, UPR dysregulation, altered lipid metabolism, and Ca^2+^ dyshomeostasis should be viewed as interconnected manifestations of progressive ER dysfunction rather than as independent pathways. Persistent activation of IRE1α–JNK and PERK–CHOP signaling promotes oxidative stress, inflammation, and mitochondrial apoptosis. In parallel, changes in ER membrane lipid composition can both modulate membrane-embedded UPR sensors and impair SERCA-mediated Ca^2+^ reuptake, thereby linking lipotoxicity to proteostatic and Ca^2+^ stress. The resulting increase in ER-to-mitochondrial Ca^2+^ transfer at MAMs may drive mitochondrial Ca^2+^ overload, bioenergetic failure, ROS production, and cytochrome *c* release. Mitochondrial ROS and ATP depletion, in turn, further impair ER protein folding, Ca^2+^ transport, and membrane homeostasis. These reciprocal interactions establish a positive-feedback loop that links persistent metabolic stress to podocyte and tubular epithelial cell injury, inflammation, and apoptosis in DKD.

### 5.5 Persistent ER stress, renal injury, and fibrosis

Persistent ER stress contributes to progressive renal injury by integrating UPR dysregulation, oxidative stress, Ca^2+^ dyshomeostasis, and mitochondrial dysfunction. In podocytes, sustained ER stress can impair cytoskeletal and slit-diaphragm integrity and promote podocyte loss, whereas in tubular epithelial cells it can disrupt protein quality control and cellular homeostasis, promoting inflammatory and apoptotic responses [80].

Persistent ER dysfunction may also promote renal fibrosis through crosstalk with profibrotic pathways. ER stress-associated oxidative stress, inflammation, and tubular injury can enhance TGF-β/small mothers against decapentaplegic (SMAD) signaling and extracellular matrix production, thereby contributing to a profibrotic microenvironment [81], [82]. ER stress can also engage innate immune signaling, as Gα12-dependent activation of IRE1α and ATF6 promotes NLRP3 inflammasome activation, pyroptosis, and renal fibrosis in DKD, an effect attenuated by Gα12 deletion [83]. Thus, ER dysfunction is not an isolated fibrotic pathway but an integrative stress response that can amplify the processes driving progressive renal remodeling and fibrosis in DKD.

## 6 Therapeutic perspectives: from experimental ER-targeted strategies to clinically established therapies

ER dysfunction represents a potential therapeutic target in DKD; however, the strength and maturity of evidence vary substantially across therapeutic approaches. Most interventions that directly target ER proteostasis, UPR signaling, lipid handling, Ca^2+^ homeostasis, or ER–mitochondrial contacts remain at the preclinical stage, whereas several established glucose-lowering therapies, particularly SGLT2 inhibitors and GLP-1RAs, have demonstrated kidney protection in clinical studies but have not been clinically validated specifically as ER-targeted therapies. Accordingly, it is important to distinguish direct experimental manipulation of ER pathways from clinically proven renoprotective treatments whose effects on ER homeostasis are supported predominantly by mechanistic and preclinical evidence.

### 6.1 Experimental ER-targeted strategies: preclinical evidence

#### 6.1.1 Restoration of ER proteostasis and modulation of the UPR

Restoring ER proteostasis represents a potential strategy for limiting maladaptive UPR signaling in DKD. Chemical chaperones such as 4-phenylbutyrate (4-PBA) and tauroursodeoxycholic acid (TUDCA) enhance protein-folding capacity and reduce ER stress. In preclinical models of diabetic nephropathy, 4-PBA reduced renal ER stress, inflammatory signaling, and proteinuria [84]. In db/db mice, 4-PBA and TUDCA decreased albuminuria and mesangial expansion and suppressed multiple ER stress markers, including BiP, phosphorylated IRE1α, phosphorylated eIF2α, CHOP, ATF6, and spliced XBP1, while reducing podocyte apoptosis [85]. TUDCA also attenuated albuminuria, tubular injury, and ER stress-associated apoptosis in diabetic mice [86]. In another diabetic model, TUDCA reduced proteinuria and podocyte and glomerular injury, accompanied by decreased ER stress signaling [80]. These findings support restoration of ER proteostasis as a potential approach to limiting downstream renal injury, although current evidence remains predominantly preclinical.

Selective modulation of individual UPR branches may provide a more targeted strategy than global ER stress inhibition. In diabetic nephropathy, pharmacological inhibition of IRE1α RNase activity with STF-083010 reduced IRE1α-associated TXNIP/NLRP3 signaling, pyroptosis, and renal injury [87]. Small-molecule approaches targeting IRE1α, PERK, or other UPR components have therefore been proposed to suppress persistent pro-inflammatory or pro-apoptotic signaling while preserving adaptive UPR activity [88], [89]. However, because the UPR is essential for maintaining ER homeostasis, complete or nonspecific inhibition may also interfere with protective stress responses. Therapeutic strategies should therefore favor selective and context-dependent modulation of maladaptive UPR signaling rather than global UPR suppression.

#### 6.1.2 Targeting ER lipid and Ca^2+^ homeostasis

Because ER lipid metabolism and Ca^2+^ handling are closely related to ER function, targeting these processes may provide a therapeutic approach for DKD. Targeting the SREBP pathway may help reduce excessive lipogenesis and renal lipid accumulation. However, studies of SREBP inhibition have shown inconsistent results. For example, fatostatin inhibited SREBP activation in diabetic kidneys but did not improve albuminuria or renal fibrosis in an experimental model of early diabetic nephropathy [89], [90], [91], [92]. These findings suggest that more selective approaches are needed to target renal lipid metabolism.

AMP-activated protein kinase (AMPK), an important regulator of cellular energy metabolism, may also help reduce lipotoxicity by inhibiting lipogenic signaling and promoting fatty acid oxidation [93]. In db/db mice, fenofibrate improved renal lipotoxicity through activation of the AMPK-PGC-1α pathway and inhibition of SREBP-1 signaling [94]**.** Although metformin is a clinically established AMPK activator, its potential effects on renal ER stress and lipid metabolism do not establish a direct ER-targeted effect. The role of AMPK activation in regulating ER dysfunction in DKD therefore requires further investigation.

Targeting Ca^2+^ dyshomeostasis is another emerging strategy. Pharmacological activation of SERCA may restore ER Ca^2+^ stores and reduce cytosolic Ca^2+^ overload. The SERCA activator CDN1163 has been shown to affect cellular energy metabolism in experimental models, although its therapeutic efficacy in DKD has not been established [95]. Overall, strategies targeting ER lipid metabolism or Ca^2+^ homeostasis remain largely preclinical and require further evaluation of renal efficacy, target specificity, and long-term safety.

#### 6.1.3 Enhancing ER quality control and ER–mitochondrial homeostasis

Enhancing ER quality control and removing damaged ER components may help maintain cellular homeostasis. mTOR inhibitors such as rapamycin can enhance autophagy and may promote the removal of damaged cellular components [96]. However, chronic systemic mTOR inhibition can affect multiple cellular processes and may cause unwanted effects, highlighting the need for more selective approaches to enhance ER quality control.

Mitofusin-2 (MFN2), an important ER–mitochondrial tethering protein, contributes to MAM integrity and mitochondrial function. In high-glucose-treated podocytes, MFN2 deficiency was associated with MAM dysfunction and apoptosis, whereas restoration of MFN2 reduced podocyte injury through the PERK pathway [97]. Recent studies have also shown that VDAC1/GRP75/IP_3_R-mediated MAM regulation is associated with MAM stability and renal injury in experimental DKD [98]. Consistent with a protective role of MAM formation, C5aR2 was recently shown to alleviate DKD by promoting PSS-MFN2 interaction and enhancing MAM formation, thereby improving ER and mitochondrial function [99]. Modulating MFN2 or other components of the ER–mitochondrial Ca^2+^ transfer machinery may therefore help restore Ca^2+^ and redox signaling between the ER and mitochondria. However, these approaches remain at an early preclinical stage, and their therapeutic potential in DKD requires further investigation.

### 6.2 SGLT2 inhibitors and GLP-1RAs: from preclinical mechanisms to clinical evidence

#### 6.2.1 Preclinical evidence for ER-related mechanisms of SGLT2 inhibitors and GLP-1RAs

SGLT2 inhibitors and GLP-1RAs have shown renoprotective effects in experimental models, and increasing evidence suggests that these effects may involve the regulation of ER stress and organelle homeostasis. In db/db mice, dapagliflozin reduced ER stress-related apoptosis, particularly through modulation of the eIF2α–ATF4–CHOP pathway [4]. In another diabetic mouse model, dapagliflozin reduced renal oxidative and ER stress, improved podocyte injury, and partly restored autophagy [5]. These findings suggest that SGLT2 inhibition may reduce renal injury by limiting maladaptive ER stress and restoring cellular quality control.

Recent studies have further linked SGLT2 inhibition to ER–mitochondrial crosstalk. In diabetic mice, empagliflozin reduced excessive MAM formation and podocyte injury, with AMPK signaling implicated In these effects [6]. Conversely, canagliflozin enhanced MAM formation and improved mitochondrial function in diabetic db/db mice, accompanied by reduced ER stress and podocyte injury [7]. These apparently divergent findings may reflect the context-dependent role of MAMs in maintaining ER-mitochondrial homeostasis. Both insufficient and excessive ER-mitochondrial coupling can be detrimental to cellular homeostasis. Insufficient coupling may impair ER-mitochondrial communication and the efficient transfer of Ca^2+^ and metabolic signals, whereas excessive or sustained coupling may promote mitochondrial Ca^2+^ overload, oxidative stress, and apoptotic signaling. Consistent with this context-dependent view, reducing MAM formation through CAP1 deficiency protects podocytes in DKD [100], whereas enhancing MAM formation through C5aR2 is also protective [99]. Therefore, the beneficial direction of MAM modulation may depend on the experimental model, cell type, disease stage, and functional state of the contact sites. Moreover, changes in MAM abundance do not necessarily correspond to changes in their functional activity. Rather than uniformly increasing or decreasing MAM formation, effective intervention may require restoring an appropriate balance of ER-mitochondrial contacts and their associated functions. These findings suggest that MAM regulation may contribute to the renoprotective effects of SGLT2 inhibitors, although the underlying mechanisms require further investigation.

GLP-1RAs may also regulate renal ER stress. In type 2 diabetic rats, liraglutide reduced renal expression of the ER stress markers GRP78 and caspase-12 and was associated with improved renal injury [8]. Overall, current preclinical evidence suggests that SGLT2 inhibitors and GLP-1RAs may influence ER stress, autophagy, and ER–mitochondrial crosstalk. However, these mechanisms have mainly been demonstrated in experimental models and have not been directly established as the cause of renal protection in patients (Table 1).

**Table 1:** Preclinical studies evaluating the effects of SGLT2 inhibitors and GLP-1RAs on ER dysfunction and renal injury.

Medication	Animal model	Dose	Duration	ER-related mechanism/outcome	Renal outcome
**Dapagliflozin**[4]	db/db mice	0.5 mg/kg/day	1 week	Reduced ER stress-mediated apoptosis, mainly involving the eIF2α–ATF4–CHOP pathway	Attenuated ER stress-related renal injury
**Dapagliflozin**[5]	HFD-induced diabetic mice	200 μg/day	8 weeks	Reduced oxidative and ER stress; partly restored autophagy and reduced HMGB1 expression	Improved podocyte injury, renal fibrosis, and urinary microalbumin
**Empagliflozin**[6]	HFD/STZ-induced diabetic mice	10 mg/kg/day	12 weeks	Reduced excessive MAM formation, with AMPK signaling implicated in these effects	Reduced albuminuria, BUN, MAM imbalance, and podocyte injury
**Canagliflozin**[7]	db/db mice	30 mg/kg/day	8 weeks	Enhanced MAM formation, accompanied by improved mitochondrial function and reduced ER stress and podocyte injury	Reduced urinary albumin excretion and podocyte/renal injury
**Liraglutide**[8]	HFD/low-dose STZ-induced type 2 diabetic rats	100 or 200 μg/kg/day	8 weeks	Reduced renal GRP78 and caspase-12 expression	Reduced serum creatinine, BUN, and 24-h urinary protein and improved renal histology

#### 6.2.2 Clinical evidence for renal protection

Clinical trials have provided substantial evidence for the renal benefits of SGLT2 inhibitors. In the EMPA-REG OUTCOME trial, empagliflozin reduced the risk of incident or worsening nephropathy in patients with type 2 diabetes and high cardiovascular risk [9]. The CREDENCE trial, which specifically enrolled patients with type 2 diabetes and diabetic kidney disease, showed that canagliflozin reduced the risk of the primary composite renal outcome, including kidney failure, doubling of serum creatinine, or death from renal or cardiovascular causes [10]. In the DAPA-CKD trial, dapagliflozin reduced the risk of sustained eGFR decline, kidney failure, or death from renal or cardiovascular causes in patients with CKD, with similar effects in participants with and without type 2 diabetes [11]. The EMPA-KIDNEY trial further showed that empagliflozin reduced the risk of kidney disease progression or cardiovascular death in a broad CKD population, including patients without diabetes [12].

Clinical evidence for GLP-1RAs has also expanded. In the LEADER trial, liraglutide reduced the risk of a composite renal outcome, although this effect was driven mainly by a reduction in new-onset persistent macroalbuminuria [13]. In the REWIND trial, dulaglutide also reduced the risk of a composite renal outcome, with the largest effect observed for new-onset macroalbuminuria [14]. More recently, the FLOW trial specifically enrolled patients with type 2 diabetes and CKD and showed that semaglutide reduced the risk of major kidney outcomes and slowed the decline in eGFR [15]. Together, these trials provide clinical evidence for renal benefits of SGLT2 inhibitors and GLP-1RAs. However, the mechanisms underlying these effects are likely multifactorial, and the contribution of ER stress and ER–mitochondrial crosstalk has not been directly established in these clinical trials (Table 2).

**Table 2:** Clinical evidence for the renal protective effects of SGLT2 inhibitors and GLP-1RAs.

Study	Study population	Intervention	Follow-up	Primary renal endpoint/renal outcome	Kidney function	Principal conclusion
**EMPA-REG OUTCOME**[9]	T2D with established cardiovascular disease	Empagliflozin 10 or 25 mg/day	3.1 years	Incident or worsening nephropathy	Reduced progression of kidney disease; slower decline in renal function	Empagliflozin reduced incident or worsening nephropathy
**CREDENCE**[10]	T2D with albuminuric CKD, n = 4401	Canagliflozin 100 mg/day	2.62 years	Kidney failure, doubling of serum creatinine, or renal/CV death	Slower eGFR decline after an initial acute decrease	Canagliflozin reduced kidney and cardiovascular events
**DAPA-CKD**[11]	CKD with or without T2D, n = 4304	Dapagliflozin 10 mg/day	2.4 years	≥50% sustained eGFR decline, kidney failure, or renal/CV death	Slower loss of kidney function	Dapagliflozin reduced the risk of the primary renal composite, with similar effects in participants with and without T2D
**EMPA-KIDNEY**[12]	CKD with or without T2D, n = 6609	Empagliflozin 10 mg/day	2.0 years	Kidney disease progression or CV death	Slower progression of CKD	Empagliflozin reduced the risk of kidney disease progression or CV death
**LEADER**[13]	T2D at high cardiovascular risk, n = 9340	Liraglutide up to 1.8 mg/day	3.84 years	Composite renal outcome	eGFR decline was slightly slower; UACR was lower	Renal benefit was mainly driven by reduced new-onset persistent macroalbuminuria
**REWIND**[14]	T2D, n = 9901	Dulaglutide 1.5 mg/week	5.4 years	New macroalbuminuria, ≥30% sustained eGFR decline, or renal replacement therapy	No statistically significant reduction in ≥30% eGFR decline; main effect on albuminuria	Reduced composite renal outcome, mainly through reduced new-onset macroalbuminuria
**FLOW**[15]	T2D with established CKD	Semaglutide 1 mg/week	3.4 years	≥50% eGFR decline, kidney failure, or renal/CV death	Slower eGFR decline and fewer major kidney events	Semaglutide reduced major kidney events and slowed kidney function loss

### 6.3 Challenges in clinical translation and future directions

Accumulating evidence supports ER dysfunction as an important component of DKD pathogenesis and provides a rationale for exploring ER-related therapeutic strategies. However, translating these mechanistic insights into precise clinical interventions will require several issues to be addressed.

First, therapeutic specificity and timing are critical considerations. ER stress and UPR activation can initially serve adaptive functions but may become maladaptive when persistent. Broad suppression of ER stress may therefore be inappropriate at certain stages of DKD. Defining disease- and cell-specific therapeutic windows and selectively targeting maladaptive UPR signaling, Ca^2+^ dysregulation, or ER–mitochondrial crosstalk may provide a more precise approach. Kidney- or cell-targeted delivery could further limit systemic effects.

Second, the relative contribution of individual ER-related pathways to renal injury remains to be defined. Current evidence supports links between ER stress, mitochondrial dysfunction, inflammation, and renal injury, but several interventions can improve these abnormalities simultaneously. Target-specific genetic or pharmacological approaches may help distinguish primary pathogenic pathways from secondary responses and identify relevant therapeutic nodes.

Third, improved biomarkers are needed to identify and monitor therapeutically relevant ER dysfunction. GRP78 and CHOP can indicate ER stress but may not fully capture its cellular origin, severity, or adaptive versus maladaptive state. Integrating ER-related markers with mitochondrial, inflammatory, metabolic, and renal injury signals may improve disease stratification and treatment monitoring, including identification of patients more likely to benefit from ER-targeted interventions.

Finally, ER-targeted strategies should be developed alongside established DKD therapies. SGLT2 inhibitors and GLP-1RAs have demonstrated renal benefits clinically, whereas their effects on ER stress and ER–mitochondrial homeostasis remain supported mainly by preclinical evidence. Future studies should determine whether selective modulation of ER dysfunction can provide additional renal protection beyond current therapies, while evaluating the long-term safety of systemic ER modulation.

## 7 Conclusion

Diabetic kidney disease is a complex metabolic disorder that can lead to progressive renal injury. ER dysfunction is an important component of this process. The ER actively converts glucotoxicity, lipotoxicity, and inflammation into cellular damage through an inappropriate unfolded protein response, disrupted lipid and calcium balance, and increased damage caused by impaired ER-mitochondrial contact. This mechanistic understanding shifts therapy towards the restoration of organelle homeostasis, employing strategies that include UPR modulators, MAM stabilizers, repurposed SGLT2 inhibitors, and GLP-1 agonists. Targeting ER dysfunction represents a promising therapeutic direction for DKD, although most mechanistic evidence remains preclinical, and its clinical efficacy and added benefit beyond current therapies require further validation.

## 8 Study limitations

Several limitations should be acknowledged. This article is a narrative review informed by a structured literature search rather than a formal systematic review; therefore, study selection was guided by relevance to the conceptual framework and was not accompanied by formal risk-of-bias assessment or quantitative synthesis. Only English-language publications were considered, and some relevant studies may have been missed because of variations in terminology or indexing practices. In addition, much of the evidence linking ER dysfunction to DKD remains preclinical, limiting direct extrapolation to human disease.

## CRediT authorship contribution statement

**Yanhong Gao:** Writing – original draft, Formal analysis. **Huijun Wu:** Writing – original draft, Formal analysis, Conceptualization. **Henglan Wu:** Software, Formal analysis. **Yinfeng Guo:** Software, Methodology. **Huanqing Gao:** Writing – review & editing, Conceptualization.

## Institutional review board statement

Not applicable.

## Data availability statement

No new data were created or analyzed in this study.

## AI tool usage declaration

The authors used ChatGPT 4.0 for grammar and spelling check to improve the readability in the writing process.

## Funding

This work was supported by the Noncommunicable Chronic Diseases-National Science and Technology Major Project (China) (No. 2023ZD0507900), the National Natural Science Foundation of China, (No. 32371194) Zhejiang Province Medical and Health Project (Grant number 2024KY1672, 2024KY437), The Jiaxing Key Discipline of Medicine-Nephrology(2023-ZC-011).

## Conflicts of interest

The authors declare no conflicts of interest.
